# Supplementary material for: Neuronal baseline shifts underlying boundary setting during free recall
Source: Nat Commun. 2017 Nov 3;8:1301. doi: 10.1038/s41467-017-01184-1 (PMC5670232; doi:10.1038/s41467-017-01184-1)
Supplement: Supplementary file 1 — Supplementary Information [file 41467_2017_1184_MOESM1_ESM.pdf]

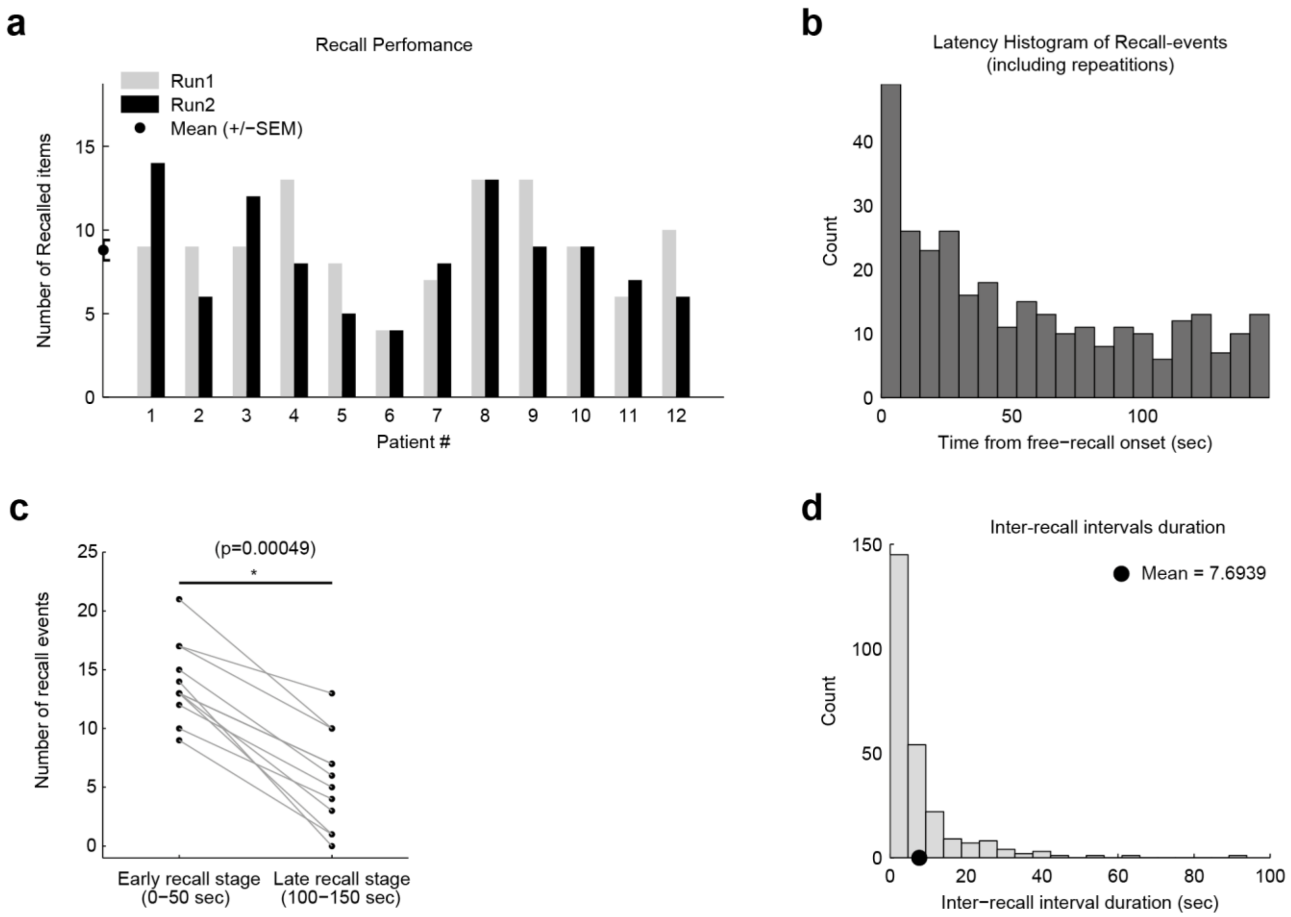

Supplementary Figure 1. Behavioral performance.

(a) Number of recalled items per run in each patient (excluding repetitions). Mean (+/- SEM): 8.79 (±0.6) items. Recall performance did not differ between the two runs ($p>0.42$, Wilcoxon signed rank test). **(b)** Time histogram showing the distribution of onsets of individual recall events (as reported verbally by the patients) during the free recall period. Note the gradual decrease in recall events over time. **(c)** Early recall stages contained significantly more recall events than late recall stages (*$p=0.0005$, $n=12$ patients, Wilcoxon signed rank test). **(d)** Histogram of inter-recall intervals, showing a right skewed distribution with fairly long inter-recall intervals (mean: 7.69 sec).

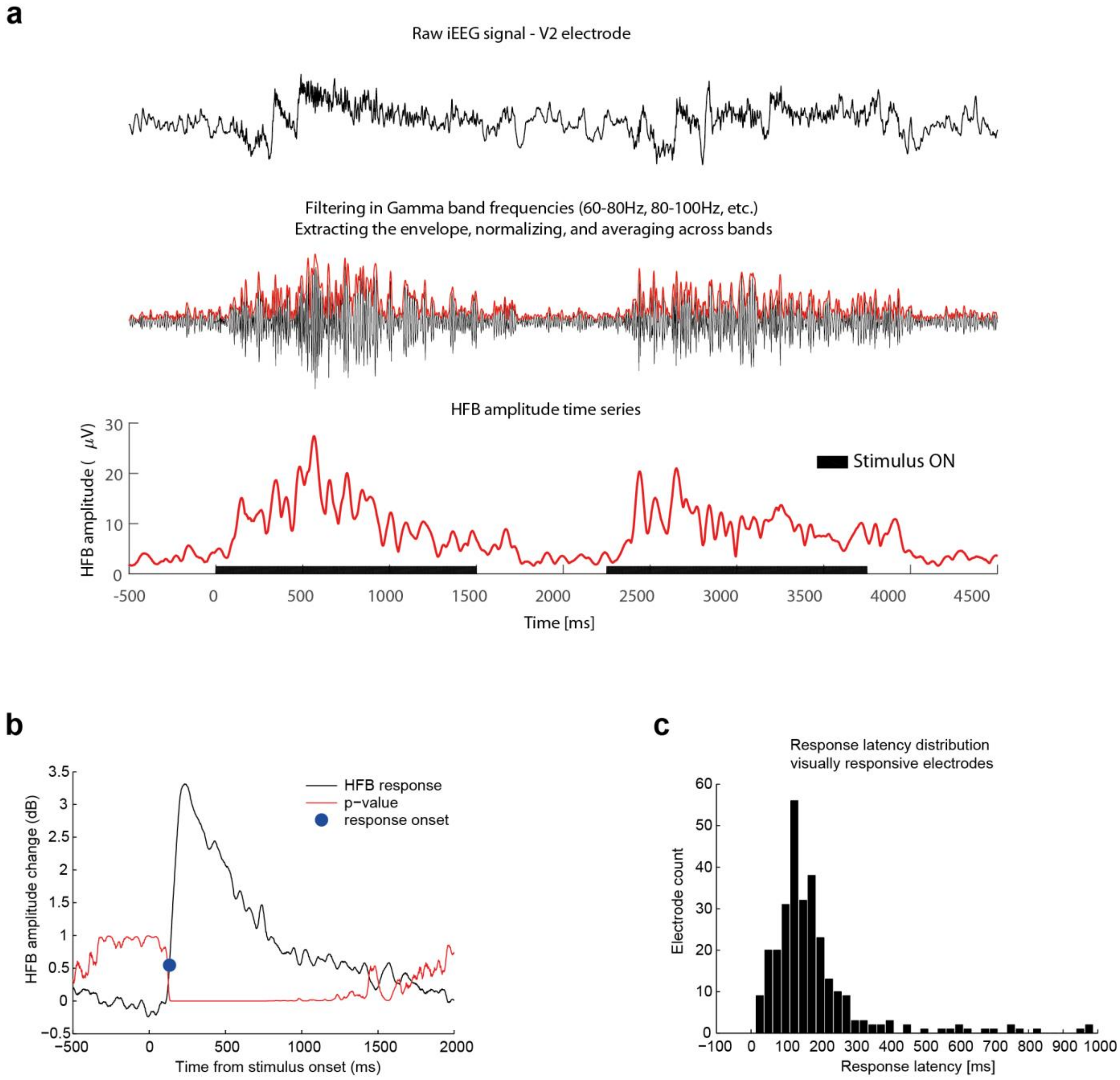

Supplementary Figure 2. HFB signal processing pipeline, an example of HFB stimulus response and distribution of response onsets across visually responsive electrodes.

(a) Top trace: raw ECoG signal during the presentation of two images. Note the high frequency activity that begins shortly after stimulus onset. Middle trace: the same ECoG signal filtered between 60-80 Hz. The envelope of the narrow-band signal (red line) was extracted by taking the absolute value of the analytic signal (obtained using a Hilbert transform). A similar procedure was done for other bands in the high-gamma range (60-80 Hz, 80-100 Hz, 100-120 Hz, etc.). The envelope of each band was normalized and then averaged across all bands to produce one HFB time series, which reflects mean neuronal population activity. **(b)** Mean HFB response to images (n=112) in one representative visually responsive electrode. Response latency was defined as the first time point in which the HFB amplitude crossed a p-value of 0.05 and remained significant for at least 50 ms. **(c)** Distribution of response latency across all visually responsive electrodes (see Supplementary Table 2 for further details).

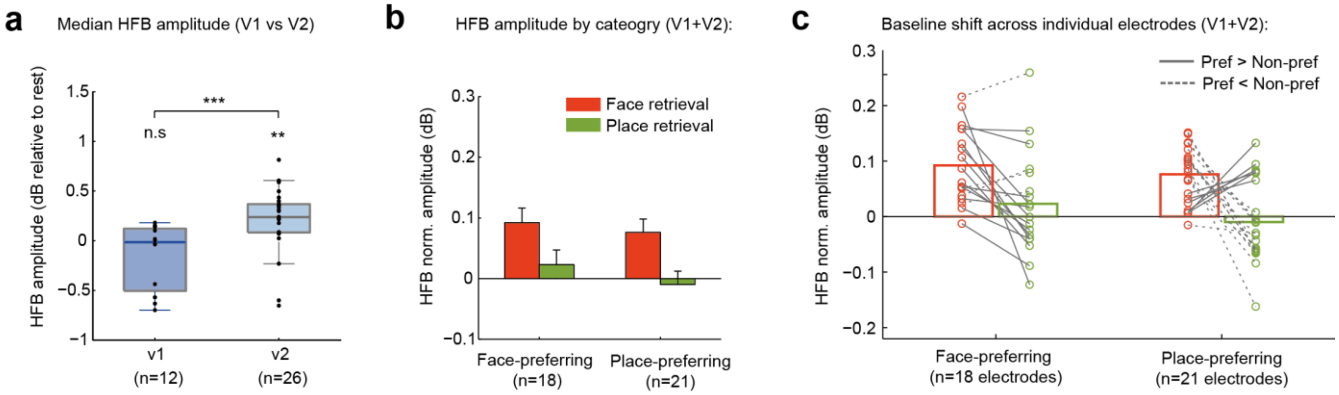

Supplementary Figure 3. HFB activity in areas V1 and V2 during the free recall period.

(a) During the free recall period electrodes in V2 showed a significant increase in median HFB amplitude relative to resting-state (**p=0.0017, Wilcoxon signed rank test). V1 electrode did not show this effect (p=0.46, Wilcoxon signed rank test). Directly comparing the two groups of electrodes using a Wilcoxon rank sum test revealed a significant difference (*** p<0.001; whiskers are 1.5 the interquartile range). **(b-c)** Early visual electrodes showed a significant difference in activity levels during the free recall period between the two categories. To check if those baseline modulations were linked to the category preference of these electrodes during viewing, we split V1 and V2 electrodes into two groups based on their category preference during viewing (face/place preference), and compared the median amplitudes during faces/places retrieval across the two groups in a two-way mixed-effects ANOVA (similar to the analysis presented in figure 3a). We did not find any relation between category preference during viewing and baseline modulation during free recall in these electrode (i.e. no interaction between ‘category-preference’ and ‘recalled-category’: F(1,37)= 0.26, p>0.6). Regardless of category preference during viewing, we found an intriguing overall increase in HFB amplitude during recall of faces compared to places (F(1,37)=22.76, p<0.0001). Error bars represent SEM.

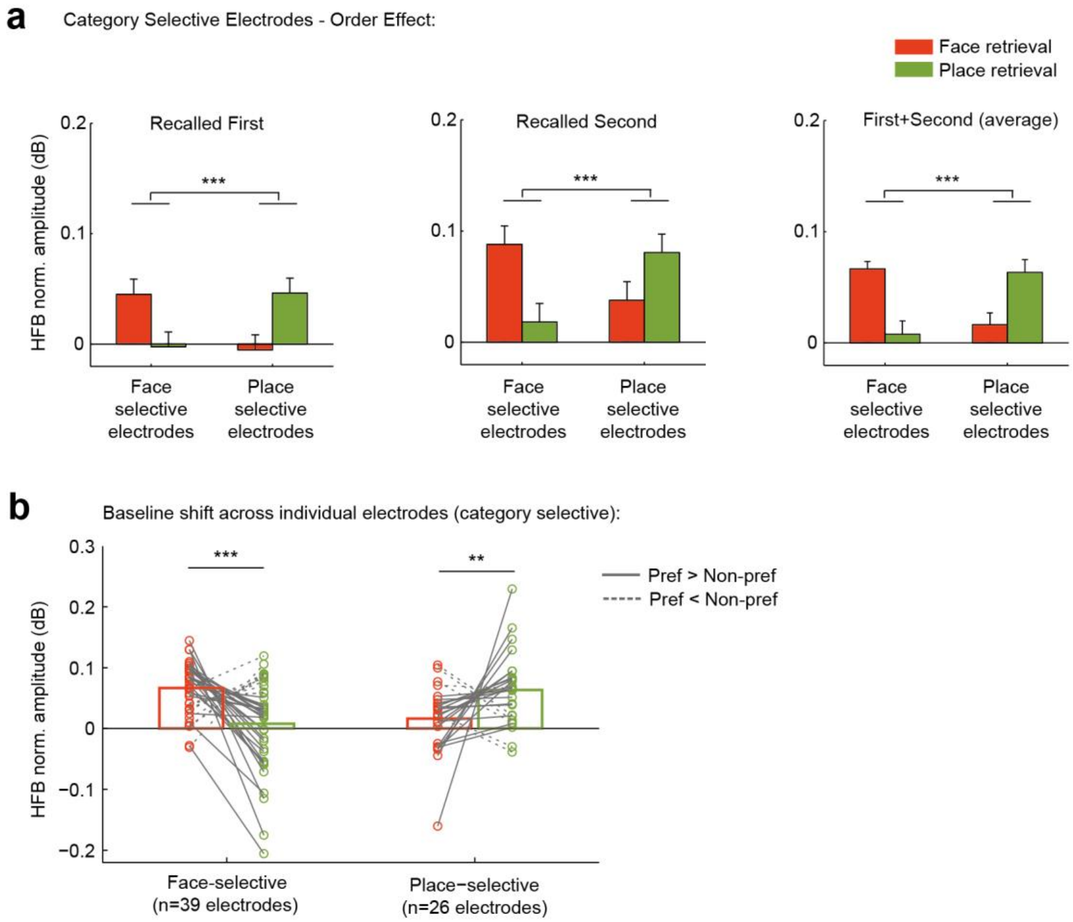

Supplementary Figure 4. Three-way mixed-design ANOVA for testing recall order effects.

(a) A mixed-design ANOVA using the fixed factors of ‘electrode group’, ‘recalled category’ and ‘recall order’, and the random factors of ‘patient’ and ‘electrode’ revealed in addition to an interaction between recalled category and electrode group, a significant recall order effect ($F(1,189)=11.10$,***$p=0.001$). A direct comparison between the two levels of recall-order (targeted first vs. targeted second) revealed an overall increase in HFB activity for both preferred and non-preferred categories when targeted second, that is, after the opposed category was already retrieved ($t(189)=-3.33$, $p<0.01$). This result is likely to reflect an increase in cognitive demands linked to the second retrieval, which is more challenging due to the temporal distance from the encoding phase and the top-down control efforts needed in order to ignore recently-retrieved items from the opposed category. **(b)** A spaghetti plot showing within electrode amplitude differences during recall, contrasting preferred and non-preferred categories (face-selective electrodes: $t(189)=4.38$, ***$p<0.0001$; place-selective electrodes: $t(189)=-2.87$, **$p<0.01$; pairwise comparisons using a mixed-effects model).

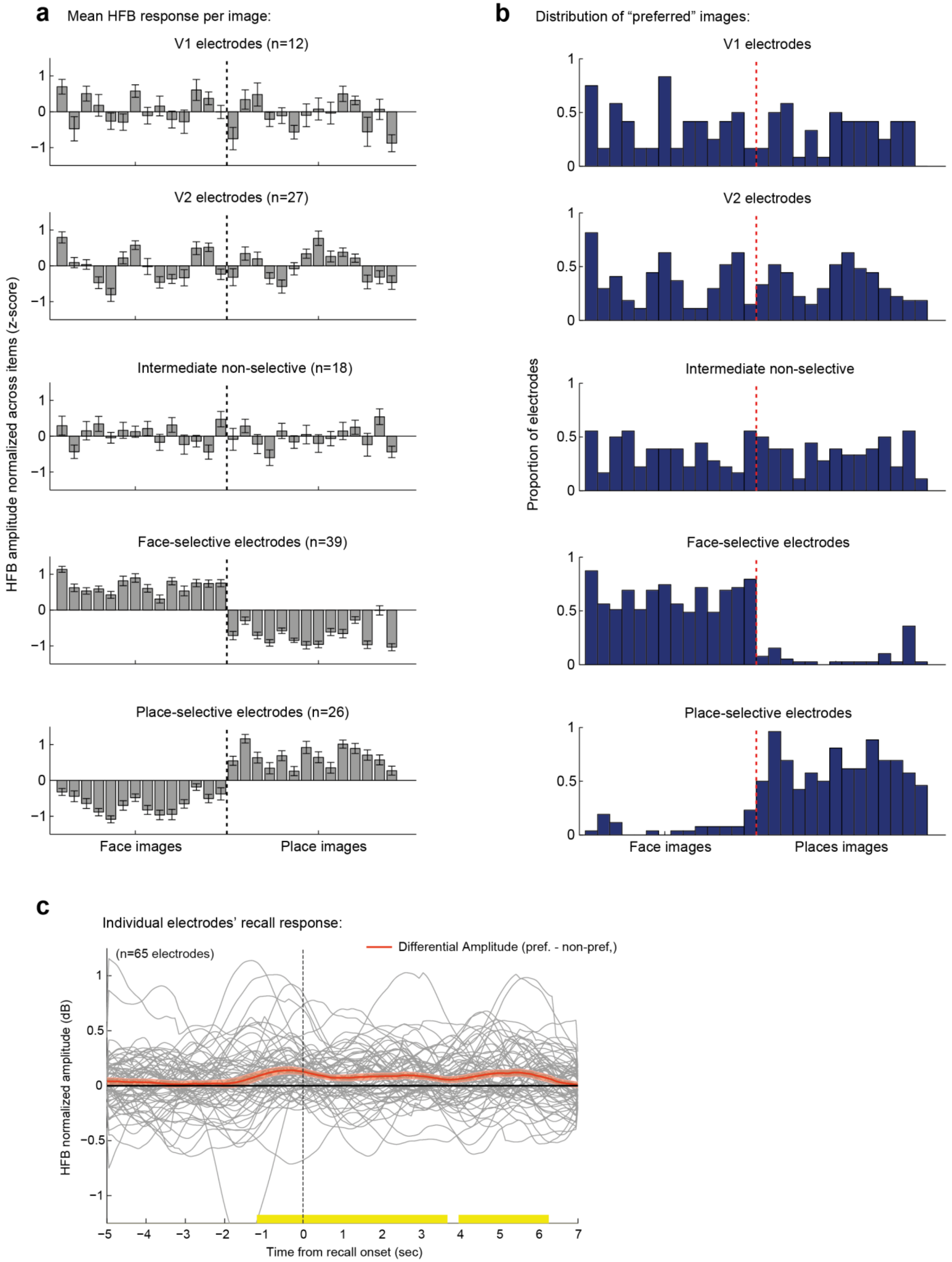
a
Mean HFB response per image:
V1 electrodes (n=12)
V2 electrodes (n=27)
Intermediate non-selective (n=18)
Face-selective electrodes (n=39)
Place-selective electrodes (n=26)
HFB amplitude normalized across items (z-score)
Face images
Place images
b
Distribution of "preferred" images:
V1 electrodes
V2 electrodes
Intermediate non-selective
Face-selective electrodes
Place-selective electrodes
Proportion of electrodes
Face images
Places images
c
Individual electrodes' recall response:
(n=65 electrodes)
Differential Amplitude (pref. - non-pref,)
HFB normalized amplitude (dB)
Time from recall onset (sec)

Supplementary Figure 5. Mean HFB response per image, distribution of preferred items across different electrode groups, and individual electrodes' response to recall events.

(a) HFB response to the presentation of each item was averaged in a time window of 100 to 500 ms post stimulus, normalized into z-score and grand-averaged across all electrodes within each electrode group. **(b)** Distribution of items defined as "preferred" (top ten responses) across the two categories. As can be seen, V1 and V2 did not show category preference, but did manifest a considerable amount of variability in the response to different exemplars. **(c)** Recall-related differential activity (preferred–non-preferred images) in individual category selective electrodes. Shaded areas represent SEM.

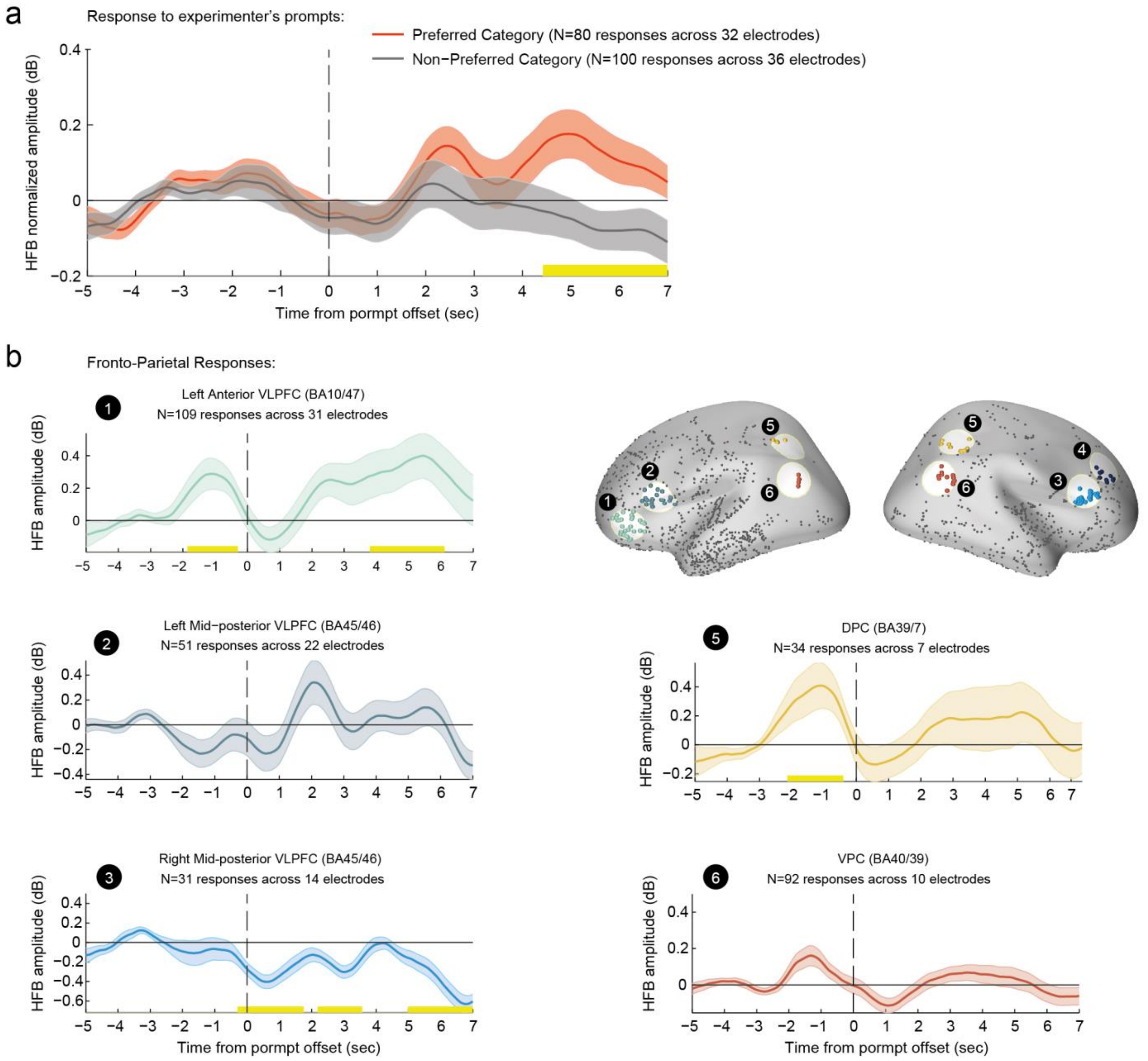

Supplementary Figure 6. Baseline shift recovery following an experimenter's prompt.

(a) HFB response to experimenter's prompts during preferred category recall (red) and non-preferred category recall (gray). Before the prompt is given there were no substantial differences between preferred and non-preferred categories. Following the prompt the baseline shift was recovered and the two signals became significantly different, with higher activity when targeting the preferred category. **(b)** HFB response to experimenter's prompts in prefrontal and parietal regions of interest. Note the significant amplitude increase in left aVLPFC and DPC. Electrodes in the DLPFC were not included in this analysis due to an insufficient number of trials. Yellow marks indicate significant time bins ($p<0.05$, cluster-based correction). Shaded areas represent SEM. Abbreviations: aVLPFC – anterior ventrolateral prefrontal cortex (~BA10/47); mid-VLPFC – mid-posterior ventrolateral prefrontal cortex (~BA45); DLPFC – dorsolateral prefrontal cortex (~BA8/9); DPC – dorsal parietal cortex (~BA39/7); VPC – ventral parietal cortex (~BA39/40).

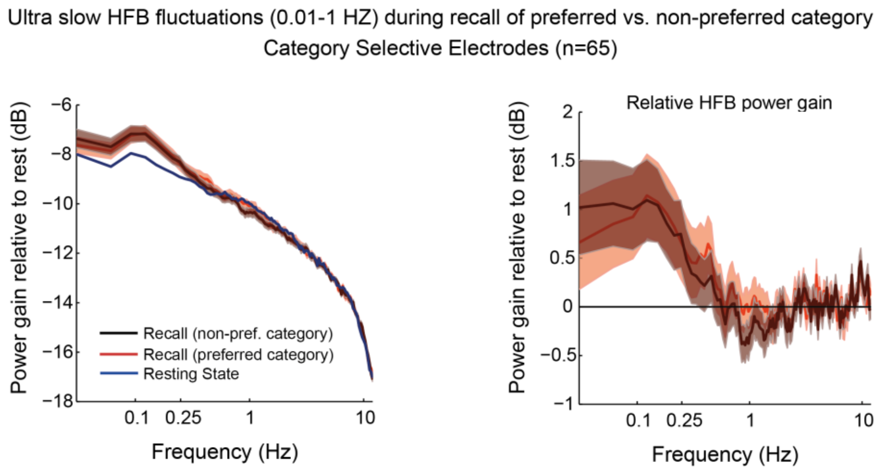

Supplementary Figure 7. Spectral analyses of ultra-slow HFB activity fluctuations (<1Hz).

Spectral analysis of HFB signals in category selective electrodes during recall of preferred vs. non-preferred category did not reveal any significant differences (p>0.05, FDR corrected, signed rank tests). Shaded areas represent SEM.

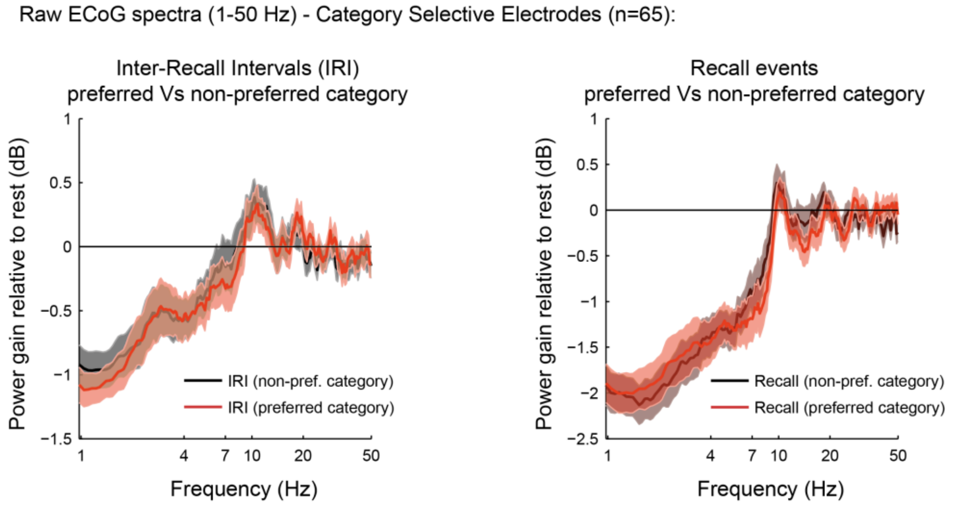

Supplementary Figure 8. Spectral analyses of raw ECoG signals (1-50Hz) in category selective electrodes during free recall of images from the preferred vs the non-preferred category.

Spectral analysis of raw ECoG signals in category selective electrodes during recall of preferred vs. non-preferred category did not reveal any significant differences ($p>0.05$, FDR corrected, signed rank tests). Shaded areas represent SEM.

Patient #	Sex	Age	Blindfold During Recall	Total analyzed electrodes	Visually responsive electrodes	Number of visually responsive electrodes in each ROI				
						Retinotopic Areas			High-Order	
						V1	V2	Intermediate non-selective	Face Selective	Place Selective
1	M	28	Closed eyes	126	29	0	4	3	8	2
2	M	50	Closed eyes	91	30	4	1	9	0	0
3	M	38	✓	83	16	0	0	0	1	3
4	M	23	✓	159	59	0	13	0	10	5
5	F	44	✓	150	19	0	4	1	4	1
6	F	29	✓	102	34	4	4	4	5	0
7	F	22	✓	225	8	0	0	0	0	0
8	F	34	✓	135	27	4	0	1	4	3
9	F	30	✓	188	14	0	0	0	4	5
10	F	29	✓	251	35	0	0	0	2	7
11	F	32	✓	238	4	0	0	0	0	0
12	F	55	✓	251	10	0	1	0	1	0
**Total**				**1999**	**280**	**12**	**27**	**18**	**39**	**26**

Supplementary Table 1. Details of patients and distribution of visually responsive electrodes across individuals.

Patient	Channel	Electrode	MNI Coordinates			Vis. Response	Cat. Selectivity	Response Latency
ID	label	group	X	Y	Z	effect size (d)	effect size (d)	(ms)
1	LGRD58	Faces	-45	-83	0	2.65	1.17	58
1	LGRD59	Faces	-38	-83	-6	1.88	1.61	70
1	LGRD52	Faces	-51	-56	-6	1.69	1.27	82
1	LTp4	Faces	-17	-40	-14	1.25	1.55	122
1	LGRD60	Faces	-33	-80	-11	1.14	4.65	98
1	LTo6	Faces	-35	-49	-24	0.93	3.65	134
1	LGRD61	Faces	-64	-55	-13	0.61	1.25	126
1	LTo7	Faces	-25	-38	-22	0.38	1.57	190
3	LTIp6	Faces	-42	-48	-16	0.80	1.59	106
4	RTp11	Faces	49	-58	-4	1.53	1.94	132
4	RTo15	Faces	61	-54	-13	1.26	0.71	32
4	RP4	Faces	18	-63	33	1.21	0.72	130
4	RTp10	Faces	49	-72	-5	1.12	1.30	118
4	RFa13	Faces	51	29	23	0.92	0.68	130
4	RP2	Faces	3	-72	27	0.72	0.95	138
4	RO12	Faces	48	-82	-4	0.65	0.92	124
4	RFo2	Faces	-7	24	-27	0.50	0.60	208
4	RTo14	Faces	60	-59	-7	0.41	0.65	80
4	RFo5	Faces	9	25	-25	0.33	0.62	202
5	Lo8	Faces	-50	-75	4	1.44	1.69	96
5	Lo9	Faces	-50	-74	7	0.88	0.90	110
5	Lo10	Faces	-49	-78	16	0.55	0.61	128
5	LTIp4	Faces	-38	-34	-20	0.42	1.10	164
6	RSOL5	Faces	28	-66	-18	0.72	0.75	150
6	RDTi1	Faces	29	-19	-34	0.72	0.89	178
6	Grid20	Faces	45	-70	14	0.59	0.88	154
6	RDTi2	Faces	33	-31	-29	0.48	0.61	182
6	Grid12	Faces	59	-60	10	0.42	0.86	134
8	RTo5	Faces	44	-50	-17	1.35	0.94	70
8	RTo6	Faces	45	-48	-16	0.78	1.75	130
8	RTp6	Faces	54	-25	-20	0.53	1.35	176
8	RTp5	Faces	56	-33	-20	0.48	0.62	170
9	LTO7	Faces	-57	-49	-25	1.14	2.77	118
9	LTp5	Faces	-30	-27	-22	0.70	0.77	162
9	Dh6	Faces	-40	-25	-20	0.58	1.06	182
9	LTm4	Faces	-42	-24	-30	0.48	1.29	196
10	LTp5	Faces	-40	-48	-19	0.89	0.91	136
10	LTp6	Faces	-43	-47	-14	0.71	0.97	150
12	LTm6	Faces	-41	-50	-24	0.45	2.11	170
1	LTo5	Places	-34	-61	-17	1.60	0.75	116
1	LTp3	Places	-14	-33	-11	0.99	0.86	122
3	LTo4	Places	-29	-54	-8	1.32	0.69	80
3	LTo5	Places	-30	-54	-8	1.23	1.05	86

3	LTo3	Places	-20	-46	-10	1.08	1.25	34
4	RTp12	Places	53	-47	-4	1.62	0.84	120
4	RO8	Places	31	-94	4	1.57	1.18	96
4	RO9	Places	36	-90	7	1.42	1.70	106
4	RFa11	Places	44	28	19	1.18	0.96	112
4	RTp4	Places	11	-56	10	0.31	0.81	400
5	LTo2	Places	-18	-37	-13	0.52	0.69	134
8	RTo3	Places	28	-45	-10	1.63	1.12	80
8	RPs7	Places	22	-65	61	0.83	0.76	124
8	RPs6	Places	22	-64	59	0.50	0.63	116
9	LTO6	Places	-33	-55	-21	2.70	1.08	108
9	LTO5	Places	-29	-54	-18	2.36	1.19	110
9	LTp6	Places	-33	-38	-21	2.03	1.38	136
9	LTp4	Places	-22	-36	-15	0.67	1.54	164
9	LTO4	Places	-28	-48	-11	0.40	0.93	122
10	RTp1	Places	14	-29	-13	1.48	1.21	96
10	RFa11	Places	52	30	19	1.33	1.86	124
10	RTp2	Places	24	-38	-13	0.66	0.77	138
10	RFi1	Places	0	25	-26	0.47	0.74	172
10	RTi10	Places	60	-42	-12	0.45	0.57	172
10	RTp3	Places	23	-34	-15	0.40	0.79	186
10	LFm11	Places	-48	41	27	0.35	0.64	214
2	RO2	V1	9	-95	-2	3.10	0.12	42
2	RO3	V1	11	-94	1	2.97	0.08	44
2	RO1	V1	11	-97	-4	2.68	0.19	54
2	RO4	V1	11	-94	-1	2.07	0.12	68
6	Grid44	V1	12	-98	-4	0.75	0.44	124
6	Grid42	V1	8	-92	-4	0.66	0.42	120
6	Grid43	V1	11	-91	8	0.61	0.35	144
6	ROi4	V1	4	-85	9	0.37	0.17	154
8	RO1	V1	5	-91	3	3.10	0.31	26
8	RO3	V1	10	-81	8	2.23	0.33	12
8	RO2	V1	8	-85	3	2.23	0.10	24
8	RO4	V1	9	-77	11	1.70	0.06	38
1	LTo1	V2	-21	-103	-14	2.73	0.12	28
1	LTo2	V2	-14	-93	-19	1.26	0.41	48
1	LTo3	V2	-9	-83	-7	0.85	0.34	44
1	LTo4	V2	-17	-77	-13	0.50	1.11	72
2	RO5	V2	28	-91	0	1.75	0.07	88
4	RO2	V2	7	-78	-11	3.05	0.13	50
4	RTo1	V2	12	-75	-9	2.88	0.24	50
4	RO1	V2	8	-77	-12	2.58	0.27	44
4	RTo2	V2	-5	-92	15	2.40	0.06	34
4	RO3	V2	12	-75	-7	2.30	0.07	40
4	RO4	V2	11	-73	-5	2.28	0.11	56

4	RO5	V2	31	-100	1	2.22	0.31	52
4	RTo3	V2	-2	-89	17	1.95	0.28	36
4	RO6	V2	30	-99	2	1.88	0.06	62
4	RO7	V2	29	-92	0	1.61	0.66	86
4	RTo4	V2	18	-57	-3	1.58	0.46	50
4	RTo9	V2	27	-90	0	0.74	0.16	80
4	RTo5	V2	16	-60	-2	0.70	0.25	66
5	Lo3	V2	-23	-89	-18	2.60	0.20	58
5	Lo1	V2	-12	-91	-10	2.32	0.53	60
5	Lo2	V2	-11	-87	-15	2.21	0.21	58
5	Lo4	V2	-20	-89	-13	1.76	0.37	78
6	Grid41	V2	9	-76	-6	0.87	0.38	114
6	ROs6	V2	4	-87	15	0.74	0.48	118
6	Grid33	V2	11	-84	-8	0.72	0.23	98
6	Grid34	V2	27	-89	-15	0.53	0.41	136
12	LTm4	V2	-16	-53	-11	0.42	1.42	156
1	LGRD51	High Ret.	-51	-80	8	1.93	0.19	74
1	LGRD50	High Ret.	-42	-88	6	1.23	0.02	100
1	LGRD49	High Ret.	-37	-87	4	0.73	0.01	N/A
2	RO6	High Ret.	29	-92	4	1.43	0.15	90
2	RTIp3	High Ret.	24	-47	-11	1.40	0.00	112
2	RTo7	High Ret.	28	-65	37	1.37	0.10	138
2	RTIp4	High Ret.	27	-51	-8	1.35	0.22	122
2	RO10	High Ret.	47	-74	3	1.32	0.02	140
2	RTIp5	High Ret.	30	-52	-8	1.27	0.14	134
2	RO11	High Ret.	45	-74	7	0.96	0.03	158
2	RTo8	High Ret.	33	-66	34	0.70	0.09	192
2	RO12	High Ret.	41	-72	9	0.63	0.30	140
5	LP6	High Ret.	-30	-70	30	0.79	0.42	114
6	Grid45	High Ret.	19	-85	36	0.75	0.48	114
6	Grid37	High Ret.	24	-82	27	0.70	0.55	96
6	Grid25	High Ret.	34	-92	-11	0.59	0.12	88
6	Grid40	High Ret.	12	-75	59	0.41	0.43	286
8	RTo2	High Ret.	22	-46	-11	0.74	0.11	84
Median (Q1-Q3)		Faces				0.71 (0.51-1.13)	0.97 (0.75-1.56)	132 (114-167)
		Places				1.13 (0.5-1.54)	0.89 (0.75-1.18)	121 (106-137)
		V1/V2				1.88 (0.74-2.35)	0.25 (0.12-0.39)	58 (43-86)
		High Ret.				0.87 (0.7-1.34)	0.12 (0.04-0.27)	114 (95-139)

Supplementary Table 2. Details of visually responsive electrodes included in the analysis, showing the approximate MNI coordinates, visual response effect size (Choen's d; comparing post-stimulus HFB amplitude [100 to 500 ms post-stimulus] to a pre-stimulus baseline [-400 to -100 ms]), category selectivity effect size (Cohen's d; HFB response to faces vs. places in a time window of 100 to 500 ms post-stimulus), and response latency (see methods). At the bottom of the table we report median and interquartile range (Q1-Q3) for each electrode group.
